# Advances in Deep Neuropathological Phenotyping of Alzheimer Disease: Past, Present, and Future

**DOI:** 10.1093/jnen/nlab122

**Published:** 2022-01-04

**Authors:** Mustafa N Shakir, Brittany N Dugger

**Affiliations:** From the Department of Pathology and Laboratory Medicine, University of California, Davis, Sacramento, California, USA; From the Department of Pathology and Laboratory Medicine, University of California, Davis, Sacramento, California, USA

**Keywords:** Alzheimer disease, Concomitant pathologies, Convolutional neural networks, Deep learning, Immunohistochemistry, Machine learning, Whole slide imaging

## Abstract

Alzheimer disease (AD) is a neurodegenerative disorder characterized pathologically by the presence of neurofibrillary tangles and amyloid beta (Aβ) plaques in the brain. The disease was first described in 1906 by Alois Alzheimer, and since then, there have been many advancements in technologies that have aided in unlocking the secrets of this devastating disease. Such advancements include improving microscopy and staining techniques, refining diagnostic criteria for the disease, and increased appreciation for disease heterogeneity both in neuroanatomic location of abnormalities as well as overlap with other brain diseases; for example, Lewy body disease and vascular dementia. Despite numerous advancements, there is still much to achieve as there is not a cure for AD and postmortem histological analyses is still the gold standard for appreciating AD neuropathologic changes. Recent technological advances such as *in-vivo* biomarkers and machine learning algorithms permit great strides in disease understanding, and pave the way for potential new therapies and precision medicine approaches. Here, we review the history of human AD neuropathology research to include the notable advancements in understanding common co-pathologies in the setting of AD, and microscopy and staining methods. We also discuss future approaches with a specific focus on deep phenotyping using machine learning.

## INTRODUCTION

Alzheimer disease (AD) is a neurodegenerative disease first described by Alois Alzheimer in 1906 in his case report “Über eine eigenartige Erkrankung der Hirnrinde” [About a peculiar disease of the cerebral cortex], which described a 51-year-old female patient presenting to clinic with thoughts of jealousy toward her husband and memory weakness (1, 2). “She then showed signs of paranoia, disorientation to time and place, and inability to perform basic tasks to care for herself (1).” His patient died 4.5 years after she was admitted to a mental asylum, and upon examining her brain postmortem, Alzheimer noted “very peculiar changes of neurofibrils are observable… Eventually, the nucleus and the cell disintegrate, and only a tangled bundle of fibrils indicates the place which had formerly been occupied by a ganglion cell” (1). Alzheimer also reported deposition of certain stainable chemical in the ganglion cells, reporting that the fibrils and the depositions seem to go hand in hand (1).

**FIGURE 1.**
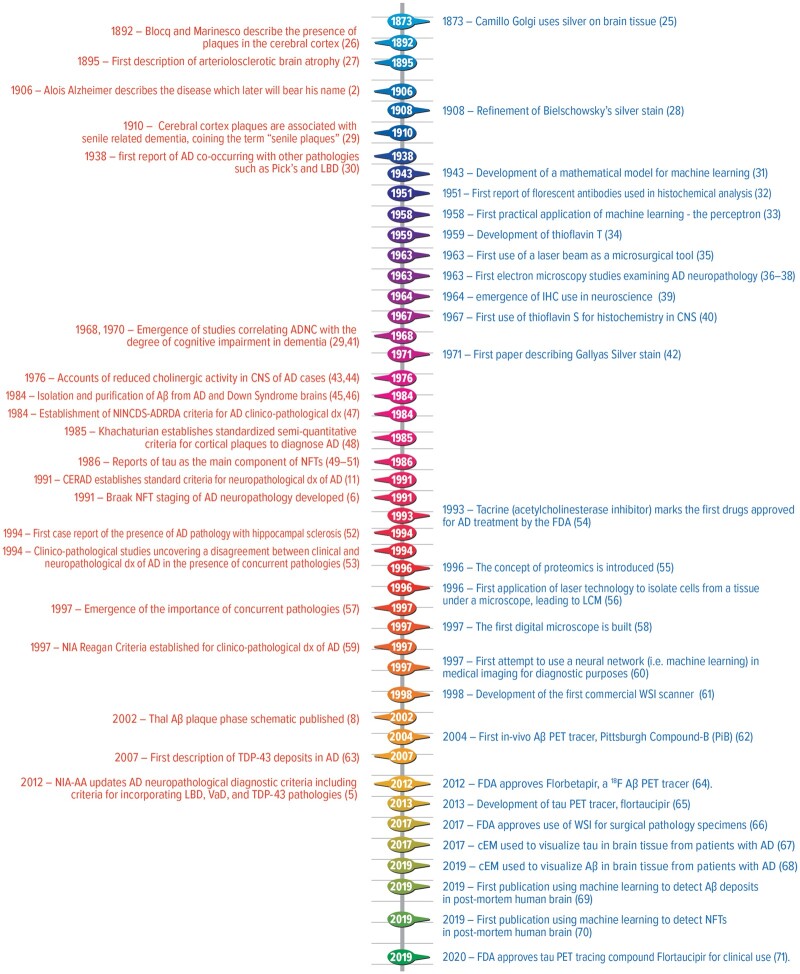
Timeline of select advancements in understanding the pathology of Alzheimer’s disease and deeper phenotyping (orange text) and select advancements in scientific tools (blue text). AD, Alzheimer disease; ADNC, Alzheimer disease neuropathologic changes; Aβ, amyloid beta; cEM, cryoelectron microscopy; CERAD, Consortium to Establish a Registry for Alzheimer’s Disease; CNS, central nervous system; dx, diagnosis; IHC, immunohistochemistry; NFT, neurofibrillary tangles; NIA, National Institute of Aging; NIA-AA, National Institute of Aging and Alzheimer’s Association; NINCDS-ADRDA, National Institute of Neurological and Communicative Diseases and Stroke/Alzheimer’s Disease and Related Disorders Association; LBD, Lewy body disease; LCM, laser capture microdissection; TDP-43, transactive response (TAR) DNA binding protein at 43 kDa; VaD, vascular dementia; WSI, whole slide imaging.

Since that case report, a plethora of publications have advanced the field of AD research. As of August 4, 2021, a simple search on PubMed for “Alzheimer disease” revealed over 173 500 peer reviewed articles on AD. Although great strides have been made (Fig.  1—timeline), the diversity of the pathophysiology processes of the disease is still not completely appreciated or understood. This review delves into the history of human AD neuropathology by examining the neuroanatomic phenotypes of AD, AD heterogeneity, and notable advancements in pathology tools, such as microscopy and staining methods that have advanced AD research. Last, we examine future direction of AD research with a specific focus on deep phenotyping using machine learning to enhance precision medicine approaches.

### AD PATHOLOGIES, LOCATIONS, AND HETEROGENEITIES

Pathological hallmarks of AD consist of the presence of extracellular aggregated amyloid beta (Aβ) protein in the form of Aβ plaques and aggregated hyperphosphorylated tau protein in the form of neurofibrillary tangles (NFTs) within the brain (3). With Aβ plaques, numerous subsets can exist based upon Aβ morphology and if the plaque is associated with dystrophic neurites (i.e. neuritic plaques) (4). These plaques and NFTs can differ in density, makeup, and anatomical distribution (4–10). Both NFTs and Aβ plaques are hypothesized to be distributed in a hierarchical fashion, with certain brain regions being more susceptible to their accumulation (3). An Aβ plaque phase and an NFT staging scheme were developed by Braak and Braak (6) and Thal et al (8), respectively, to denote neuroanatomical progression. Furthermore, a semiquantitative assessment has been developed through the Consortium to Establish a Registry for Alzheimer’s Disease (CERAD) to denote neuritic plaque burden present during neuropathological analysis (11). These systems are incorporated into the current neuropathological diagnostic guidelines for AD put forth by the National Institute on Aging and Alzheimer’s Association (NIA-AA) (5, 7).

For Thal Aβ plaque phase, the process starts in the neocortex (designated as “Phase 1”), slowly progressing inferiorly through the diencephalon and brainstem, eventually ending in the cerebellar cortex (designated as “Phase 5”) (5, 8). For Braak NFT stages, the process starts at the level of the transentorhinal cortex (termed transentorhinal Stages I and II), moves to the limbic region (Stages III and IV), and finally reaches the association isocortices (Stages V and VI) (6). Additional works hypothesize that tau deposits may occur earlier in areas other than the entorhinal cortices, such as the locus coeruleus (12, 13) and the olfactory bulb and tract (14). Some researchers place greater emphasis on tau deposits in neurites (i.e. neuropil threads) when assessing disease progression (15). To calculate the CERAD neuritic plaques score, semiquantitative assessments are conducted in the densest square millimeter area of neocortex (including superior/middle temporal gyri, middle frontal gyrus, and/or inferior parietal lobule) (11). The scale range is none (no neuritic plaques present), sparse (>0 but <6 neuritic plaques), moderate (between 6 and 20 neuritic plaques), or frequent (having >20 neuritic plaques) (11).

Following these staging/phasing processes, an ABC score is then derived- “A” for Thal amyloid phase, “B” for Braak NFT staging, and “C” for CERAD neuritic plaque densities. This ABC score is then further synthesized to denote the likelihood of AD neuropathologic changes (ADNC) contributing to the clinical presentation of the patient (Not, Low, Intermediate, or High) (5, 7). Unlike previous consensus criteria, such as NIA-Reagan, classifications are independent from clinic presentation of the patient (5, 7). This is significant because, in certain instances, patients can have ADNC and be clinically asymptomatic (5, 7, 16). In addition, some groups have further stratified AD to include “asymptomatic at-risk for AD,” “presymptomatic AD,” and then “prodromal (i.e. preclinical) AD” (17), further showing the clinical heterogeneity and complexity of the pathophysiology.

Although the spatial dispersion pattern of Aβ plaques and NFTs is often predictable, there are instances in which the distribution of these pathologies has been variable and did not follow the previously mentioned schematics. These cases have taken on identities such as atypical AD (18). Two neuropathologically defined subtypes of atypical AD have arisen: the hippocampal sparing subtype (18), in which the hippocampus is relatively spared from degeneration relative to the rest of the cortex (19), and the limbic-predominant type AD (18), in which NFT deposition is severe but restricted to the medial temporal lobe relative to the rest of the cortex (20). Furthermore, there have been attempts to classify AD into different subtypes *in-vivo* using positron emission tomography (PET) imaging patterns (21). For example, Vogel et al (21) proposed subtyping AD into a limbic subtype, a posterior occipitotemporal subtype, a medial temporal lobe sparing subtype, and a temporal lateral subtype. In addition, there have been intriguing studies relating to primary age-related tauopathy (PART) (22). PART is a recently coined term used to describe the presence of NFTs in brains with minimal or no Aβ plaques, and hence are currently not on the continuum of the AD spectrum (22). PART can be very common among older individuals, regardless of the presence of clinical dementia (22). Previous terms used to describe tauopathy-related changes were either not distinctive enough between clinical and pathological diagnosis, or used pejorative terms like “senile” (22). The transition in language to PART was made to reduce the use of pejorative terminology and to align these changes with the NIA-AA revised diagnostic criteria that separate the clinical presentation from the neuropathological presentation (22).

In the context of the above-mentioned variances of AD, it becomes clear that during postmortem examination, robust, quantitative analyses of the neuroanatomic distribution of pathologies are important to establish deeper phenotyping of AD, and to aid in creating—as one may state—a deeper neuropathologic landscape. Moreover, analyzing the neuroanatomic distribution of AD pathologies can provide insights into potential concomitant pathologies such as Lewy bodies (23, 24). For example, Dugger et al (23) revealed in clinicopathologically diagnosed Lewy body disease (LBD) cases with probable rapid eye movement sleep behavior disorder had a lower Braak NFT stage and were less likely to have frequent neuritic plaques present. Furthermore, within the current recommendations for clinical and pathological diagnosis of dementia with Lewy body (DLB), the degree of ADNC observed (Braak NFT stage) influences the likelihood that Lewy-related pathology findings are associated with a typical DLB clinical syndrome (24).

Although we have focused on hallmark pathologies in this review, other pathophysiological changes have been noted to occur in AD including neuronal and synaptic loss, and inflammatory processes such as those related to microglia and astrocytes (3, 72). The role of inflammation in AD is not fully elucidated, and there are data to support both helpful and harmful roles that may change based on temporal aspects of disease as well as based on the abundance and type of inflammatory proteins/cells (72). Additional history of AD, especially the early stages related to defining its etiology has been reviewed previously (73).

## CONCOMITANT PATHOLOGIES WITHIN AD

**FIGURE 2.**
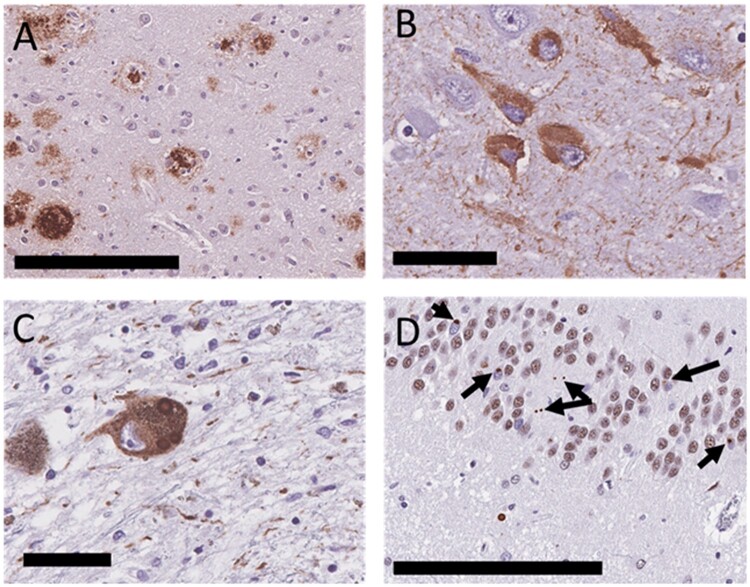
Types of pathologies seen in the setting of Alzheimer disease (AD); AD is defined by the deposition of **(A)** Aβ plaques and **(B)** neurofibrillary tangles, furthermore Lewy type synucleinopathy, such as Lewy bodies and Lewy neurites **(C)** and TAR DNA binding protein at 43 kDa (TDP-43) deposits (black arrows, using an unphosphorylated antibody to TDP-43) **(D)** can also be located within the setting of AD. Scale bars: B, C = 50 µm; A, D = 200 µm.

**FIGURE 3.**
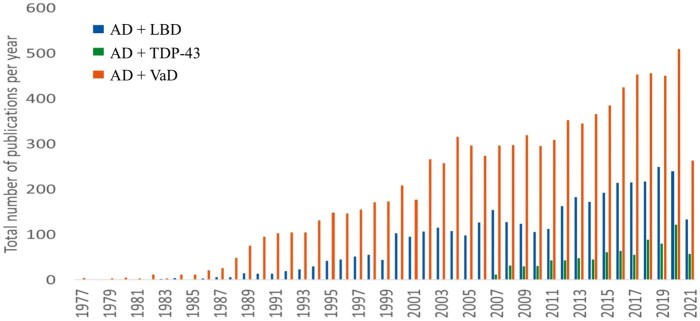
Number of articles published each year looking at “Lewy body dementia” and “Alzheimer disease” or “vascular dementia” and “Alzheimer disease” or “TDP-43” and “Alzheimer disease” (query done on August 4, 2021) Query was done using Medical Subject Headings (MeSH) headings and Used for Terms through the Ovid database. To compare the history, discovery, and prevalence of Alzheimer disease heterogeneity. LBD, Lewy body dementia; VaD, vascular dementia.

Although AD is the most common cause of dementia worldwide (74), “pure” AD (i.e. a clinical diagnosis of dementia and upon postmortem analysis the brain contains only AD pathologies, not meeting any other criteria for another neuroclinicopathological diagnosis) is not as frequent (75). Cases often have other neurodegenerative comorbidities within the setting of AD (75–81). The most common concomitant pathological diagnoses found in the setting of AD are LBD, deposits of TAR DNA binding protein at 43 kDa (TDP-43), and vascular dementia (VaD) (76–78, 80, 81); these pathologies can exist in multiple combinations and severities (82, 83). Some examples of these pathologies are shown in Figure  2. Figure  3 graphs the number of articles published each year since 1977 using the terms “Lewy body dementia” and “Alzheimer disease” or “vascular dementia” and “Alzheimer disease” or “TDP-43” and “Alzheimer disease” in a PubMed query on August 4, 2021.

It is unknown whether these concomitant pathologies are caused by each other and/or are the results of a common underlying pathophysiological change (84). It does seem, however, these pathologies may have some synergistic effects on each other, as seen in the case of some vascular pathologies and AD, as well as TDP-43/LBD-AD (85–87). The coexistence of these pathologies is very common, with the first reported copathology of AD and LBD dating back to 1938 (30). Interestingly, Henrich Lewy and Alois Alzheimer worked in the same laboratory making disease overlap even more intriguing. Synergistic effects can be clinically significant as some studies have shown that concomitant pathologies can alter the threshold of dementia diagnoses (87). In a clinical setting, these diverse underlying pathophysiologies make diagnosis a challenge. Furthermore, it is not just a “who” or “what” (i.e. what pathologies) but a “when” and “where,” as the temporal and neuroanatomical aspects of these processes may also alter clinical presentations. Multiple studies have shown the clinical diagnostic accuracy of AD alone is not at 100% when compared with the neuropathological diagnosis, with some studies reporting full accordance of AD clinical and pathological diagnoses in 49% of their cohort (88). This study reported a sensitivity of 46% and a specificity of 88% when it came to diagnosing AD with at least one other neurodegenerative comorbidity (88). Beach et al (89), examining 900 individuals National Alzheimer’s Coordinating Center database, revealed sensitivity ranged from 70.9% to 87.3%, whereas specificity ranged from 44.3% to 70.8% for probable/possible AD levels of clinical confidence compared with AD neuropathological diagnoses. Kovacs et al (78) encourage diagnostic procedures should not be terminated after finding the most obvious neuropathological change in the demented brain due to the common existence of multiple neurodegenerative disorders. Furthermore, much of the work on diagnostic accuracy has been done on cohorts consisting of predominately upper-middle-class White Caucasians, and may not capture the diversity of our world. This is important as many persons from backgrounds other than non-Hispanic white can have increases in mixed pathologies (90, 91). Specifically, a recent study examining Latino decedents revealed the clinical diagnosis of AD to have 97.1% sensitivity and 57.9% specificity for autopsy-verified AD (92). There is an increasing need for more diverse cohorts in neuropathology studies to understand the variety of neuropathologic landscapes to assure adequate treatment, diagnosis, and prognosis for all individuals who suffer from AD and related dementias.

## AD AND LBD

LBD is a neurodegenerative disorder characterized by accumulation of aggregated α-synuclein in the brain, leading to the development of Lewy bodies (found within neuronal cytoplasm), and Lewy neurites (found in neuronal processes) (3, 93). Clinically, LBD can present as DLB, Parkinson disease dementia, or Parkinson disease (94). In 1997, α-synuclein, a presynaptic protein, was revealed to be the main component of Lewy pathologies (95). Similar to AD, LBD has a predictable neuroanatomic distribution of α-synuclein deposits, and there have been an evolution of staging systems to assess the pattern of the distribution. The first reported staging scheme by Kosaka et al (96) was in 1984 that included 3 categories of brainstem, transitional, and diffuse LBD. Nearly a decade later in 2003, Braak et al (97) provided a finer-grained 6-stage system and used a semiquantitative approach, and although stated, the staging procedure proposed did not require evaluation of lesional density. Then in 2009, Beach et al (98) refined things further to be more inclusive of the LBD spectrum, including subjects with more limbic-predominant Lewy pathologies, with the Unified Staging System using the semi-quantitative scoring proposed by McKeith et al in the third DLB consortium. In 2021, the latest staging system was reported by Attems et al (99), termed the Lewy Pathology Consensus Criteria, and focused on a dichotomous scoring approach with a justification of minimizing interrater reliability. However, this dichotomous approach may result in inflated reporting of LBD diagnoses. The DLB Consortium has also put forth clinico-neuropathologic recommendations with a semi-quantitative approach for Lewy pathologies, with the first consensus report in 1996 (100) and the most recent fourth consensus report published in 2017 (24).

There have been studies that hypothesized the presence of subtypes within LBD, differing in certain clinical features and/or anatomic distributions (23). LBD commonly exists with other pathologies, including AD and other neurodegenerative diseases (23, 79, 101). Different subtypes of LBD, based on the anatomic location of Lewy bodies, tend to be more commonly present with AD pathology, with the most common being amygdala predominant (102–104). With amygdala predominant LBD, studies have shown Lewy bodies to positively correlate with NFTs but not Aβ plaques (105). This has led researchers to hypothesize the presence of LBD pathologies in the amygdala in the setting of AD to be a distinct form of α-synucleinopathy (105, 106). These subtypes, as well as the presence of co-pathologies, have caused alteration in the most recent consensus criteria for LBD, reflecting the importance of the anatomic distribution of Lewy bodies and incorporating ADNC when assessing the likelihood of associations with clinical syndromes (24). This new incorporation is significant because the overlap between AD and LBD can be highly prevalent; depending on the cohort examined, up to 50% of brains with AD pathology also show LBD pathology (79, 104, 107). Some have even termed this common occurrence as “triple-brain amyloidosis” (consisting of α-synuclein, NFTs consisting of tau proteins, and Aβ plaques of Aβ aggregates) (108). The presence of LBD in addition to AD is clinically significant because the course of the AD-LBD disease tends to be more aggressive and with more pronounced cognitive dysfunction than pure AD (23, 109–111).

## AD AND TDP-43

TDP-43 is normally a nuclear protein involved in mRNA processing; it was not until 2006 that TDP-43 was implicated in the pathogenesis of some neurodegenerative diseases (112). TDP-43 is associated with a subset of frontotemporal lobe dementias, amyotrophic lateral sclerosis, and hippocampal sclerosis (112). Hippocampal sclerosis has been associated with AD since 1989, when Zweig et al (113) reported the presence of AD pathology in patients with hippocampal sclerosis; Dickson et al (52) later confirmed this finding with a larger study. Although TDP-43 can be a *sine qua non* for select diseases, it can also be present within the setting of other diseases, such as AD, but does not necessarily indicate the presence of hippocampal sclerosis plus AD (3, 114, 115). The first report of concomitant TDP-43 and AD was in 2007, and presence of TDP-43 proteinopathy in the setting of AD can be somewhat common, ranging from 20% to 37% (63, 80, 116). Studies examining the rate of TDP-43 deposits in individuals with AD or at risk of AD (i.e. people with Trisomy 21) found that brains with ADNC had higher rate of TDP-43 deposits than those not showing ADNC, suggesting a potential common association between the neuropathological origin of these diseases (117–120). Similar to LBD, the presence of TDP-43 in the setting of AD has been noted to enhance cognitive impairment, represented by reduced scores on cognitive function tests, as well as increased likelihood of developing AD-like symptoms before death (80, 86, 121). Certain areas of the brain tend to harbor ADNC and TDP-43 pathology concurrently, particularly the amygdala, entorhinal cortex, and dentate gyrus of the hippocampus (63, 122–125). There also have been TDP-43 staging schemes based on the deposition in these select anatomic areas (115, 126, 127), with limbic-predominant TDP-43 encephalopathy being the most recent terminology proposed; albeit there is still much to understand regarding the spectrum of TDP-43 deposition in human brain (115, 128).

## AD AND VAD

In contrast to the former proteinopathies, VaD is not defined by intracellular pathogenic protein accumulation, but rather is associated with cerebrovascular disease, which occurs on a spectrum of intracranial vessel injuries denoted by infarctions, hemorrhages (129, 130), and other vascular pathologies such as cerebral amyloid angiopathy (87, 131) and arteriolosclerosis (132). Although all forms of cerebrovascular pathology have the potential to increase risk of dementia, VaD seems to be most correlated with pathology involving the microvasculature of the brain, termed small vessel disease (133). Historically speaking, the association between brain vascular pathology and the distinct type of dementia it causes (e.g. different than “senile dementia,” which was the term assigned to AD dementia) has been appreciated and noticed even before AD, by Alois Alzheimer himself (27).

The clinical presentation of AD-associated dementia is different than VaD-associated dementia. The former is insidious and slow in onset, whereas the latter is abrupt and predictable in onset and progression (134). However, it can be difficult to clinically ascertain the difference between VaD and mixed VaD-AD dementia (134). As with LBD and TDP-43, VaD is prevalent with AD, presenting in over 30% of cases (87, 133, 135), and is associated with greater cognitive impairment than pure AD (87, 133). Furthermore, concomitant AD and VaD have been noted to be more frequent in select ethnoracial groups, especially those of Hispanic descent (91). As for staging schemes and creating a consensus for diagnosing VaD, there have been multiple attempts (136–140); however, there is no universally used system in place.

## STAINING AND MICROSCOPY ADVANCEMENTS AND RELATIONS TO AD

When it comes to making a neuropathologic diagnosis of AD, ADNC must be observed on histological analysis of the brain (7). Hence, postmortem analysis of brain tissue remains the gold standard for a definitive diagnosis of the disease (141). Generally speaking, histological analysis of postmortem samples involves fixing and cutting the tissue, staining the tissue for visualization/detection of the different structures within, and finally analyzing the stained specimen for pathologic changes utilizing a microscope. Below we review select advancements in these methods related to diagnoses as well as deeper phenotyping of the disease.

## ADVANCES IN STAINING

In terms of AD, the current standard method of evaluation is when formalin-fixed paraffin-embedded sections are subjected to immunohistochemistry (IHC) using antibodies for select proteins such as Aβ and tau. Although the main pillars of neuropathological findings in AD have been known for decades (see timeline for first discovery of Aβ plaques and NFTs components), the road to reaching a consensus on the diagnostic process has been complicated, as diagnostic techniques have evolved and changed dramatically since the disease was first reported, over a century ago.

In his initial report, Alois Alzheimer used a modified version of the silver stain developed by Bielschowsky to visualize plaques and NFTs (2, 142). Bielschowsky’s silver method was one of the many iterations of the silver stain, which was originally developed by Camilo Golgi in 1873, who used silver to stain cellular components (25). A few years after that, Ramon y Cajal modified the stain to visualize deeper structures within a neuron (for review, see [143]). Cajal’s stain was further developed to the mirror reaction silver stain, which was the basis for the Bielschowsky’s method (for review see [144]). Bielschowsky’s modification allowed for the amount and size of the silver precipitate to increase, thus allowing for better tissue visualization (144). Although this staining technique allowed Dr. Alzheimer to visualize the plaques and NFTs within the tissue, full appreciation of plaques and NFTs was still a challenge.

Another pivotal advancement in staining was in 1942, when florescent antibodies revealed pneumococcal antigen in tissues, this marked the first published report to our knowledge of using antibodies for diagnostic purposes (145). The use of antibodies in histology allowed one to “tag” a particular antigen of interest, to facilitate better localization and visualization of said antigen. In 1951, Coons (32) incorporated this new florescent antibody technique into histochemical analysis, marking the first reported use of IHC in medicine, to our knowledge. About 15 years after, IHC began to be incorporated into studying the nervous system, when Rauch et al (39) used the technique to attempt to isolate a protein in the spinal cord. Later on in 1986, this technique played a major role in determining the core component of NFTs, which reacted positively to antibodies that target tau protein (45, 49). Presently, there are a plethora of antibodies available to visualize select species of tau and Aβ to denote pathologies and their progression (for reviews, see [4, 146, 147]).

During IHC’s infancy, there were further efforts focused on advancing the staining techniques to improve tissue visualization. This led to the discovery of the thioflavin stains (thioflavin T and S), which were discovered in 1959 and 1967, respectively (34, 40). These 2 stains were similar as they revealed aggregates that exhibit beta-pleated sheet structures or amyloid structures and differed in the light emission they exhibit under fluorescence microscopy (34, 40). In addition to the discovery of thioflavin stains, the silver stain was further optimized later on in 1971 by Gallyas (42). This stain, which took his name, can selectively stain different parts of the cell, depending on the initial chemical preparations of the stain (144) as well as select tau species/isoforms. Bielschowsky’s method can be used complementary to Gallyas, as the former reveals predominately senile plaques and 3-repeat tau deposits (Pick bodies), and the latter revealing 4-repeat tau deposits (144). The Gallyas stain was used by Braak and Braak (6) to develop the NFT staging scheme.

## ADVANCES IN MICROSCOPY

Besides advancements in staining, the evolution of microscopy has also played a pivotal role in AD phenotyping and understanding of the underlying pathophysiology. Back in 1906, Alois Alzheimer used an optical microscope to visualize the neuronal changes that occurred in his patient (1, 142). Since then, advancements in microscopy such as the use of electron microscopy (EM) and cryoelectron microscopy (cEM) as well as the development of light-sheet fluorescence microscopy (LSFM), laser capture microdissection (LCM), and virtual microscopy (with whole slide imaging [WSI] in 1997; see timeline) have expanded our ability to visualize tissue changes at a much greater detail. EM allows one to visualize structures at a much stronger magnification (up to 300 million times, compared with 1500 on a light microscope, allowing observations at an atomic level) than the ordinary optical microscope (148), and the first use of EM in AD dates back to 1963 when a study examined the cortex/white matter of 5 patients with a presenile dementia diagnosis (36). cEM allows the visualization of biomolecular structures at a very high resolution, and was recently used in 2017 and 2019 to visualize tau and Aβ secondary protein structure after purification from brains of patients with AD (67, 68). Furthermore, a recent cEM paper provides evidence for a hierarchical classification of tauopathies that complements neuropathology and clinical diagnosis (149). cEM is an exciting area as it has the capabilities of providing a better understanding of the structure of pathological proteins shedding further light on disease pathophysiology (67, 68, 150, 151). LSFM was developed in 1993 and was termed orthogonal-plane fluorescence optical sectioning (152); later, it was optimized to the current LSFM in 2004 (153). This microscopy technique allows one to acquire images at a much faster rate than the typical microscope allows. LCM was first applied in 1996, 30 years after development of the first laser that could perform microsurgery (i.e. isolating parts of tissue without damaging the surroundings) (35). This advancement in microscopy allows one to isolate very small sections of tissue (as small as a single cell) under a microscope, allowing for a more precise and isolated observation of the tissue undergoing examination (56). Last, WSI was first developed in 1997 and gave one the ability to transform glass slide sections into digital images, improving quality, resolution, and ease to visualization (for review see [61]). The technology has been used broadly across many fields including (but not limited to): teleconsulting, archiving, research, pathology, and even education (154). For example, some medical students are starting to learn pathology using WSI in the United States (155). WSI was approved by the Food and Drug Administration (FDA) for use in surgical pathology in 2017, making it a potential key player in histopathological diagnostics (66). WSI has advantages over traditional microscopy such as the ability to partition digital images into pixels and sort them by hue/light/saturation and/or red/green/blue values for more quantitative approaches and percent areas affected by select staining. Following WSI, images can be viewed through a computer interface, eliminating the need for a microscope and enhancing the ability to share images for collaborative, consultation, and educational purposes. There are disadvantages as well, WSI can produce a variety of formats (SVS, bigtiff, CZI, etc.) due to the vastly different scanners on the market and a lack of universal format for image production (156–159), scanning systems can differ in the exposure and color contrast they can offer as well as associated metadata and compression rates; some allow changes in contrast/exposure at acquisition only, whereas others allow it later (154). Another issue associated with WSI is large file sizes, a single digitized slide may produce a file of 1 GB in size, and with numerous slides/cases across multiple studies, storage/transferring of images become important items to consider when developing the infrastructure necessary to support the technology. Despite the disadvantages, WSIs are potential key player in contributing to the neuropathologic landscape providing deeper phenotyping of AD neuropathologic landscape (18, 23, 160–162).

## OTHER ADVANCEMENTS

Other scientific advancements such as the development of the field of proteomics (and other “omic” entities), the discovery of *in-vivo* PET imaging biomarkers for AD pathologies, as well as the emergence of technologies such as digital spatial profiling (DSP) and machine learning algorithms have aided with deeper phenotyping of AD. Proteomics is defined as the study of proteins and their processes, and this field has been crucial in AD research, as the pathology of the disease is mainly driven by a proteinopathy. Proteomics was first established in 1996, following the discoveries of genome sequencing in the 1970s, 2D gel electrophoresis to separate proteins in 1975, and antigen retrieval from paraffin-embedded tissues in 1992 (55). Another advancement was in biomarkers; Pittsburgh Compound-B was first discovered in 2004 (62) and was based on concepts of the thioflavin stains for detecting amyloid structures. It gave clinicians and researchers the ability to visualize Aβ *in-vivo* using PET imaging (62). An ^18^F compound with a longer half-life, Florbetapir, was approved by the federal FDA for clinical use in 2012 (64). In 2013, an ^18^F tau tracer, Flortaucipir, was developed for *in-vivo* examination of tau deposits on PET imaging (65) and was FDA approved in 2020 for use in a clinical setting (71). There have also been other PET ligands for tau and Aβ and are reviewed elsewhere (163, 164). Select PET biomarkers have been shown to be moderately sensitive/specific for diagnosing AD compared with the gold standard neuropathological diagnosis. Beach et al (165) found Florbetapir had between 69% and 95% sensitivity and between 83% and 89% specificity (depending on the reader of the scan) in diagnosing AD. The increasing accuracy of these *in-vivo* techniques has led researchers to an *in-vivo* biomarker classification system for AD, termed the “A/T/N classification system” (166). This system proposes classifying AD *in-vivo* through a variety of imaging techniques (i.e. PET, MRI) and fluid biomarkers (i.e. CSF protein biomarkers of AD) that measure Aβ (A), tau (T), and degree of neurodegeneration (N) (166).

Finally, within the past decade, use of technologies such as DSP and machine learning for postmortem brain tissue analysis has emerged for deeper phenotyping of AD (69, 70, 167, 168). DSP aims to perform spatial profiling of highly multiplex proteins or RNAs during postmortem IHC analysis (169). In the medical literature, DSP has been mostly used to study RNA and protein spatial interactions in cancer (169, 170). Prior to this technology, the ability to spatially profile proteins was limited in their multiplexing and quantification (169). DSP is an important technology as it can aid in understanding how a protein spatially behaves in an environment with other proteins giving further insight into disease pathophysiology (169). As for machine learning, the next section will provide an overview of the technology, how it has been applied medically, with a specific focus on AD, and current cautions to consider.

## THE FUTURE OF AD DEEP PHENOTYPING USING MACHINE LEARNING TOOLS

**FIGURE 4.**
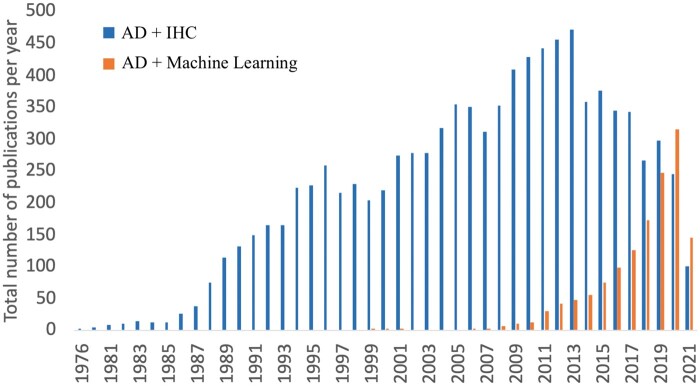
Number of articles published each year containing the search items “immunohistochemistry” and “Alzheimer disease” or “Machine learning” and “Alzheimer disease” (query done on August 4, 2021). Query was done using MeSH headings and used for terms through the Ovid database to compare the history and prevalence of immunohistochemistry relative to machine learning. AD, Alzheimer’s disease; IHC, immunohistochemistry.

Attempts at correlating a specific clinical phenotype of a patient with AD pathology have been ongoing since 1968 (29, 41), and the current neuropathologic diagnostic criteria for AD are still not robustly quantitative (5). A scalable quantitative method to characterize AD could aid immensely in deeper phenotyping of disease, and one immerging methodology that may provide a scalable, reproducible option is machine learning. To illustrate the rise of machine learning in the field, comparison to that of say IHC, Figure  4 depicts the number of articles published each year containing the search items “immunohistochemistry” and “Alzheimer disease” or “machine learning” and “Alzheimer disease” on PubMed.

Machine learning, which can be broadly defined as the discipline of teaching computers how to learn patterns from a dataset, has recently been applied to the medical field, as experts are observing that some clinical data are appropriate for machine learning (171). The association between machine learning and neurology dates back to 1943, when a theoretical mathematical framework was developed to frame neuronal activity in terms of a calculus model (31). In terms of general practical application, machine learning was first put to use in 1958, when a model was developed to examine information storage and organization in the brain (33). In the past few years, the application of machine learning to disease etiology and presentation has gained traction, as it has reached a level equivalent to that of an expert pathologist when it comes to cancer diagnoses, particularly skin and breast cancer (172–174). Specifically, a class of machine learning named convolutional neural networks (CNNs) has been at the forefront of medical diagnosis, especially for tasks that rely heavily on visual inspection (e.g. examining digital slides) (69, 175). CNN models can learn to extract patterns from visual data (i.e. WSI) after adequate training without the need for an operator to manually define image parameters or working templates (175). For further details regarding machine learning and CNNs in the general context of pathology please see the recent review by Rashidi et al (175).

In 2019, machine learning was applied in the neuropathology realm, and analysis of WSI has shown promising results as algorithms were able to precisely identify tauopathies and reach expert level AD pathological quantification (70). With respect to Aβ pathologies, a ground breaking study outlined a pipeline using WSI that produces machine learning derived scores comparable to the semiquantitative scores given by an expert pathologist (69); this pipeline has since been validated in independent cohorts (167). These machine learning methods paired with WSI could help reduce the interrater variability seen with using the CERAD criteria for AD diagnosis (176), as well as provide a scalable means to increase our capability of deeper phenotyping of AD, further improving our understanding of the disease. CNN algorithms have also been developed to classify different topographical distribution of tau pathology in progressive supranuclear palsy and corticobasal degeneration (177, 178). Additionally, there have been works to determine whether CNNs can help classify AD *in-vivo* using neuroimaging data; 1 study revealed the use of CNN and traditional machine learning yielded an accuracy rate of 98.8% for classifying AD and 83.7% for predicting the transition from mild cognitive impairment to AD (179).

A specific class of CNN models named U-NET, is particularly effective at analyzing WSI in segments (i.e. image segmentation) (180), which can prove to be helpful in determining the regional differences of AD pathology deposition (181). U-NET is loosely defined as a fully functioning CNN, and it was first described in 2017 (182). The model has been primarily used to quantify and detect cancer morphology on WSI, showing a moderately high detection rate of 75% for urothelial cell carcinoma (183). It has also been compared with expert pathologist opinion in detecting and quantifying immune cells in certain cancers, and has shown a moderately high agreement score with the pathologist evaluation (184). In terms of AD, the model has been used to aid in gray matter and white matter segmentation (181); and Wurts et al (185) have recently hypothesized that a pretrained U-NET model may be successful at identifying and segmenting tau pathologies in AD.

Although machine learning will aid immensely with scalable deep phenotyping of AD, it has not yet reached a point where it can replace a neuropathologist. The technology should be used to augment the ability of the neuropathologist, especially as the number of pathologists in the field has been decreasing; machine learning is intended to be a part of this “clinical decision support system” (186). Machine learning does have certain cautions to consider before algorithms reach a point of mass application. Namely, the robustness of CNNs across larger diverse cohorts without making major adjustments to the pipeline remains one of the largest concerns in the field (167). So far, machine learning analysis of WSI has been conducted in select cohorts only (i.e. a health system or research study cohort), on select anatomic areas with select IHC stains so these pipelines may fail when utilizing other samples unless major adjustments are made (167). This is understandable as machine learning algorithms, such as CNNs, require training data that come from the cohort they are applied to, so when there is a change in the cohort, the CNNs “training” must be adjusted as well. Some groups have been successful in developing workflows applicable to different cohorts with minimal modifications, paving the way for more general and all-around robust pipelines to be developed (167). Another hurdle is the bias-variance tradeoff that is seen in supervised machine learning, which is the most common type used in pathology (175). The bias-variance tradeoff states that if the dataset the CNN is given is very specific, then the predictive value of the CNN will be high, but the application of the algorithm will be limited (175). On the other hand, if the dataset given is very general, the predictive value of the model will be low, but the algorithm will be applicable to a wider range of datasets (175). This also can add to the risk of overfitting, which is the idea that the training dataset may become too broad and start to fit nonpathological data into the model, giving erroneous results. To overcome this hurdle, a CNN model that effectively balances the variance with the bias is needed to establish an algorithm that is both highly predictive and applicable to multiple datasets. Furthermore, machine learning in the realm of pathology relies heavily on WSI, and WSIs have many limitations (discussed in advancements in microscopy); due to the nature of this dependence, all of the limitations of WSI become limitations in applying machine learning (187). These application limitations are still being discovered, for example, it was recently revealed that the format of the imaging (i.e. PNG vs JPEG) fed into the machine learning model did not impact the performance of the model (188). Although this may not be the case for all variables, further research is needed on how these pre-analytical variables can influence the performance of a model (188). Appropriate infrastructure also needs to be considered in order to encompass the entire workflow from slide scanning to implementing machine learning algorithms. This infrastructure should include not just equipment (slide scanner, data storage/servers, and graphic processing units—either cloud-based or on premises) and space but personnel with appropriate expertise, including pathologists, machine learning engineers, IT personnel, statisticians, and database managers to aid in proper development and maintenance of the workflow. Multidisciplinary dialogs are key in the future of this technology, and will become critical in its application.

The field of machine learning is, in many ways, still at its infancy. The use of the technology is expanding, and despite all the limitations mentioned, machine learning continues to gain popularity in the scientific and medical communities. A 2020 review identified 64 machine learning-based medical devices and algorithms that are FDA approved (189). Furthermore, the FDA has acknowledged the potential impact of the machine learning as future medical devices, and published a discussion paper with propositions on how to manage machine learning-related devices in terms of policy and safety (190). Additionally, some groups have already developed, clinically validated, and experimentally implemented a machine learning model for the diagnosis of prostate cancer in a routine clinical practice (191). To further promote these technologies and have more generalizability, in the field of cancer research, there have been recent efforts toward establishing an open-access digital database for histological slides to be shared and made public (192). This is an important step for machine learning, which is dependent on data sharing to develop valid and reliable algorithms. An open-access database means the ability to develop a pipeline algorithm that can learn from data that are broader and more diverse both on the levels of cohort demographics and disease spectrum.

## CONCLUSION

**FIGURE 5.**
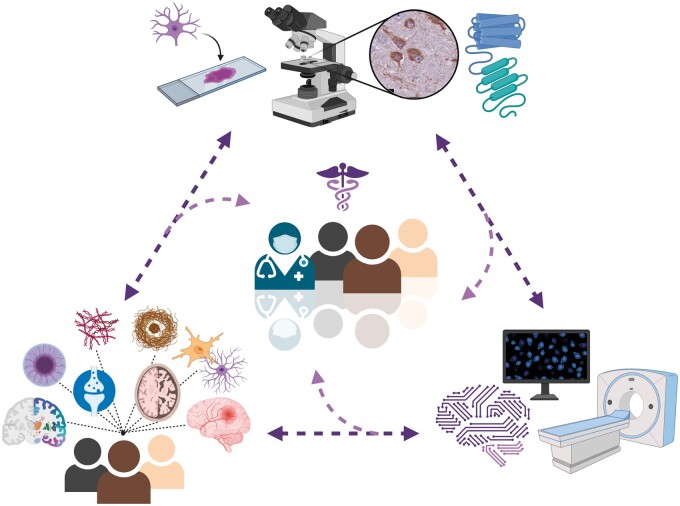
Visual representation of the concept of Alzheimer disease deep phenotyping to aid in precision medicine approaches. Heterogeneity of the disease (ie. the neuropathologic landscape) is illustrated by the diversity of the affected population and pathological phenotypes (bottom left). Microscopy, staining, and structural studies (top) as well as additional technologies (bottom right) including *in-vivo* positron emission tomography and machine learning algorithms are valuable tools for enhanced characterization. The arrows illustrate the synergetic interconnectedness of all aspects to create the deep phenotype leading to precision medicine. Figure created with BioRender.com.

Over the past century, there have been significant advancements in AD neuropathology research that have improved our understanding of its pathophysiology. The advances bring into focus the heterogenous nature of the AD neuropathologic landscape. In addition, research is moving to more robustly quantitative methods, using techniques such as machine learning to provide deeper disease phenotyping to allow for viewing of the neuropathologic landscape in a scalable way. This progression is extremely important in the journey to develop effective therapies and improved prognosis and diagnosis for AD for all individuals. Specifically, having a better phenotyping and understanding of AD is a necessary step to adapting a precision medicine approach to treating the disease (193). Precision medicine is a treatment and prevention approach that takes into account the unique presentation of each patient and tailors therapies to match the patient’s specific circumstances that lead to the diseased state (193). The concept of AD deep phenotyping to aid in precision medicine approaches should take into account phenotype heterogeneity examining data from persons of diverse backgrounds and incorporate scientific tools that enhance understanding of disease pathophysiology (overview in Fig.  5). Precision medicine is used for certain cancer therapies, and the approach is starting to make its way into the neurodegenerative realm of medicine. The concept of integrating machine learning with precision medicine is not new as both fields of radiology and oncology have integrated machine learning algorithms into their practice to personalize therapies for their patients (194, 195). When it comes to AD, similar to machine learning integration, the integration of precision medicine into the field is still at its beginning. In fact, less than 2 years ago, Alzheimer Precision Medicine Initiative was started (196). This is an international organization that aims to implement nascent AD therapies based on a patient’s unique biomarkers, genetic makeup, and disease state (196). Machine learning and precision medicine have already begun to intersect in the field of AD, and with the appropriate effort and technology, these new advancements may bring us closer to curing and preventing AD. 

## Notes

This work was supported by the National Institute On Aging of the National Institutes of Health under Award Number AG062517, a research grant from the University of California Office of the President (MRI-19-599956), a medical student research fellowship from UC Davis School of Medicine, and supported by the California Department of Publinc Health Alzheimer's Disease Program (Grant # 19-10611) with partial funding from the 2019 California Budget Act. The views and opinions expressed in this manuscript are those of the author and do not necessarily reflect the official policy or position of any public health agency of California or of the United States government.
